# High resolution melting analysis of the *18S* rRNA gene for the rapid diagnosis of bovine babesiosis

**DOI:** 10.1186/s13071-019-3781-4

**Published:** 2019-11-06

**Authors:** Jinming Wang, Aihong Liu, Shangdi Zhang, Shandian Gao, Muhammad Rashid, Youquan Li, Junlong Liu, Quanying Ma, Zhi Li, Zhijie Liu, Jianxun Luo, Guiquan Guan, Hong Yin

**Affiliations:** 1 State Key Laboratory of Veterinary Etiological Biology, Key Laboratory of Veterinary Parasitology of Gansu Province, Lanzhou Veterinary Research Institute, Chinese Academy of Agricultural Sciences, Xujiaping 1, Lanzhou, 730046 Gansu People’s Republic of China; 2 Department of Clinical Laboratory, The Second Hospital of Lanzhou University, Lanzhou, 730000 Gansu People’s Republic of China; 3 Jiangsu Co-Innovation Center for the Prevention and Control of Important Animal Infectious Disease and Zoonosis, Yangzhou University, Yangzhou, 225009 People’s Republic of China

**Keywords:** Real-time PCR, High resolution melting analysis, Diagnosis, Bovine babesiosis, *18S* rRNA

## Abstract

**Background:**

Bovine babesiosis is caused by protozoan parasites of the genus *Babesia* and presents a wide spectrum of clinical manifestations. Disease severity depends on the type of *Babesia* species infection. Generally, *B. bovis* and *B. bigemina* are considered as the causative agents of bovine babesiosis; in addition, *Babesia ovata* and *B. major* are a group of benign bovine piroplasms. Therefore, species identification is important for diagnosis, epidemiological investigations and follow-up management.

**Methods:**

Real-time PCR combined with high resolution melting (RT-PCR-HRM) analysis was used to detect and discriminate four *Babesia* species infective to cattle, including *Babesia bovis*, *B. bigemina*, *B. major* and *B. ovata*. The melting profiles and melting temperatures (Tm) of the amplicon targeting *18S* rRNA revealed differences that can discriminate the four *Babesia* spp. Sensitivity and specificity of the analytical method were evaluated using 50 blood samples collected from experimentally infected cattle and 240 blood samples from areas where bovine babesiosis is an issue.

**Results:**

RT-PCR-HRM analysis allowed to detect and discriminate four *Babesia* spp. (*B. bovis*, *B. bigemina*, *B. major* and *B. ovata*), which were responsible for bovine babesiosis in China. The protocol was validated with DNA samples from experimentally infected cattle and field infection in cattle.

**Conclusions:**

Our results indicate that RT-PCR-HRM is a fast and robust tool for the simultaneous detection and discrimination of four *Babesia* species that are responsible for bovine babesiosis in China. This approach is applicable for both field and experimental samples, thus it could be useful in epidemiological investigations and diagnoses of bovine babesiosis.

## Background

The protozoan parasites of the genus *Babesia* (phylum Apicomplexa, order Piroplasmida), cause a large spectrum of clinical manifestations known as babesiosis. This disease is a public health burden to humans and domesticated and wild animals in tropical and subtropical regions of the world. To date, more than 100 *Babesia* species have been identified in wild and domestic animals across the world [1]. However, only a few *Babesia* species have been identified in bovines and cause bovine babesiosis, namely *Babesia bovis*, *B. bigemina*, *B. major*, *B. divergens*, *B. ovata*, *B. orientalis*, *B. occultans* and *B. jakimivi.*

In China, bovine babesiosis was first reported as early as 1948 [2]. Shortly thereafter, *B. bigemina* and *B. bovis* were identified in 22 and 16 provinces, respectively [3–8]. Six *Babesia* species strains infective to bovines have been isolated by the Vectors and Vector-Borne Diseases (VVBD) Laboratory, Lanzhou Veterinary Research Institute (LVRI). These *Babesia* spp. were identified as four distinct species, *B. bovis*, *B. bigemina*, *B. major* and *B. ovata*, which were isolated from cattle in bovine babesiosis domestic areas in China [9–12].

Disease severity depends on the host’s immune status and the species of *Babesia*. Generally, *B. bovis* and *B. bigemina* are considered the causative agents of bovine babesiosis [13]. While the clinical signs caused by these parasites are similar, characterized by fever, anemia and even death, more negative effects are observed in cattle infected with *B. bovis* than *B. bigemina* [14]. *Babesia ovata* and *B. major* are a group of benign bovine piroplasms [15]. Thus, species identification is essential for diagnosis, follow-up management and epidemiological studies.

Traditionally, microscopy, considered to be the gold standard for babesiosis confirmation, is used to detect *Babesia* species in peripheral blood smears [16]. However, this approach requires skilled personnel and has several disadvantages, which include low sensitivity and unreliability in species identification [17, 18]. Recently, polymerase chain reaction (PCR) based methods (e.g. restriction fragment length polymorphism, reverse line blot, nested-PCR and multiplex PCR) are predominately used to detect and distinguish *Babesia* species with high sensitivity, specificity and repeatability [8, 19–21]. However, these assays are labor-intensive and require multiple step manipulation of the PCR product in order to determine the specific species [22]. Thus, a rapid, efficient and reliable diagnostic approach is essential for species identification.

The high-resolution melting (HRM) assay is a novel and powerful molecular method applied to mutation detection, genotype analysis and species identification [23–26]. During double strand DNA (dsDNA) dissociation to single stranded DNA with increasing temperature, melting curve and melting temperature (Tm) are generated by monitoring the fluorescence of the binding dye present in dsDNA [27]. The changes in melting curve shape and Tm peaks are generated due to differences in amplicon composition. Currently, there are no reports on HRM assays for discrimination of *Babesia* species infective to bovines. However, assays have been performed to identify *Babesia caballi* and *Theileria equi*, discriminate four *Babeisa* sp. infective to humans and diagnose five *Plasmodium* spp. [27–29]. In the present study, we developed a reliable, rapid and powerful RT-PCR-HRM assay targeting the *18S* rRNA gene to discriminate between four *Babesia* spp. in cattle.

## Methods

### Plasmid DNA preparation

Positive blood samples for each of *B. bovis*, *B. bigemina*, *B. major*, *B. ovata*, *Theileria annulata*, *Theileria orientalis*, *Theileria sinensis* and *Anaplasma marginale* were provided by the Vectors and Vector-Borne Diseases (VVBD) Laboratory, Lanzhou Veterinary Research Institute (LVRI). Total DNA was extracted from 200 μl of blood sample using a commercial DNA extraction kit (QIAamp DNA Blood Mini Kit; Qiagen, Hilden, Germany) according to the manufacturerʼs instructions. Negative control DNA was isolated from the whole blood of piroplasm-free cattle, which was examined by blood smear microscopy and nested PCR [30]. The extracted DNA was stored at −20 °C until required.

*18S* rRNA gene was amplified from DNA samples positive for *B. bovis*, *B. bigemina*, *B. major* and *B. ovata* using the primer pair (Piro1-S: 5′-CTT GAC GGT AGG GTA TTG GC-3′ and Piro3-AS: 5′-CCT TCC TTT AAG TGA TAA GGT TCA C-3′) [30]. A 25 μl PCR reaction mixture was prepared containing 2.5 μl of 10× PCR buffer (Mg^2+^ plus), 2.0 μl of dNTPs (2.5 mM each), 1.25 U of *Taq* DNA polymerase (TaKaRa, Dalian, China), 2.0 μl of template DNA, 1.0 μl of each primer (10 pM) and 16.25 μl of double-distilled water. PCR reactions parameters were as follows: initial denaturation at 95 °C for 3 min; 35 cycles of denaturation at 95 °C for 30 s, annealing 55 °C for 30 s and extension at 72 °C for 90 s; with a final extension step at 72 °C for 5 min.

PCR amplicons from each *Babesia* species were purified and cloned into a pGEM-T vector using the Easy Vector System (Promega, Madison, WI, USA). Briefly, PCR amplicons were purified with a Zymoclean™ Gel DNA Recovery Kit (Zymo, Los Angeles, USA), cloned into a PGEM-T Easy vector (Promega) and then transformed into *Escherichia coli* DH5α competent cells. Three clones of each sample were selected, prepared and sequenced using BigDye Terminator Mix (Genscript, Nanjing, China). Sequences of *18S* rRNA gene from *B. bovis*, *B. bigemina*, *B. major* and *B. ovata* were subjected to Blast analysis on the NCBI website using the BLASTn program (http://www.ncbi.nlm.nih.gov).

### Experimentally infected and field-collected samples

Six cattle were purchased from Wuwei county of Gansu Province and were confirmed to be free of piroplasm infection by the microscopy, nested PCR and ELISA assay [30–33]. Three cattle were inoculated intravenously using 10 ml of cryopreserved blood infected with *B. bovis.* The rest were injected with *B. bigemina-*infected blood. After inoculation, weekly blood samples were collected for DNA extraction.

From August 2008 to July 2014, 240 blood samples were randomly collected into EDTA coated tubes from cattle distributed across ten counties, across seven provinces in China [34]. All blood samples were transported to the VVBD, LVRI, Chinese Academy of Agricultural Sciences (CAAS) in iceboxes and stored at −20 °C. DNA extractions were performed according to the manufacturers’ instructions above and the concentration evaluated using a Nano Drop 2000 spectrophotometer (Thermo Fisher Scientific, MA, USA). All samples were initially detected using a nested PCR assay [30, 31]. From these results, the field samples were composed of 71 cases of *Babesia* spp. infections, 33 cases of *B. bovis*, 20 cases of *B. bigemina*, five cases of *B. ovata*, four cases of *B. major*, 7 cases of co-infection with two species (3 cases of *B. bovis* + *B. bigemina*; 2 cases of *B. bovis* + *B. ovata*; 1 case of *B. bigemina* + *B. ovata*; 1 case of *B. bovis* + *B. major* and 2 cases of triple co-infection (*B. bovis* + *B. bigemina* + *B. ovata* and *B. bovis* + *B. bigemina* + *B. major*).

### Real-time PCR high resolution melting analysis (RT-PCR-HRM)

HRM assays were performed using a Rotor-Gene Q6000 Real-Time PCR system (Qiagen, Sydney, Australia). Each reaction was performed in a total volume of 20 μl containing 10 μl of Forget-Me-Not™ qPCR Master Mix (Biotium, Fremont, USA), 2 pmol of each primer (BovisB-7F: 5′-CCT GAC ACA GGG AGG TAG TGA CAAG-3′ and BovisB-7R: 5′-GGC TGC TGG CAC CAG ACT TGC CCT CC-3′), 1 μl (10 ng) plasmid DNA bearing the *18S* rRNA gene sequence. The reaction parameters were as follows: initial denaturation at 95 °C for 2 min, followed by 40 cycles of denaturation at 95 °C for 5 s and annealing at 60 °C for 30 s. After real-time PCR, HRM was performed from 75 °C to 90 °C rising by 0.2 °C with a 2 s hold time at each acquisition step. Finally, melting curves were normalized using the High Resolution Melt software v.2.3.1 (Qiagen).

### Evaluation of sensitivity and specificity of the RT-PCR-HRM

To determine the analytical sensitivity and detection limits of the HRM assay, 10-fold serial dilutions of positive control plasmid DNA were used as template, ranging from 10^7^ to 1 copy number/μl. Each plasmid dilution was duplicated in three independent experiments to ensure reproducibility of the threshold cycle number (Cq). Standard curves were plotted using the software v.2.3.1 (Qiagen) to evaluate the amplified efficiency percentages and linear correlations of each *Babesia* species.

The specificity of the HRM analysis was also determined using genomic DNA extracted from bovines and parasites infective for bovines, including *T. annulata*, *T. orientalis*, *T. sinensis* and *Anaplasma marginale*.

## Results

### Polymorphic regions on the *18S* rRNA gene and primer design

**Fig. 1:**
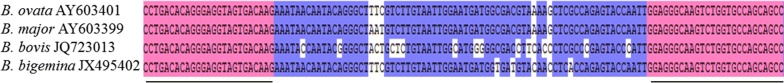
Nucleotide sequences of the *18S* rRNA amplicon and the primer location. Sequence alignment of the nucleotide sequence of the *Babesia* spp. amplicon used in the HRM analysis. The underlined sequences indicate the position of the primer pair used for the real-time PCR assay

Sequences for the *18S* rRNA gene of piroplasms infective for bovines were retrieved from GenBank (accession numbers JQ723013 for *B. bovis*, JX495402 for *B. bigemina*, AY603399 for *B. major* and AY603401 for *B. ovata*). After sequence alignment using DNAMAN v.2.0 software, conserved and variable regions were identified for each *Babesia* species. A primer pair was designed for the conserved regions to amplify variable gene fragment using the Primer v.5.0 software (Fig. 1). A 122-bp amplicon was amplified using the primer pair Bovis-B-7F and Bovis-B-7R; the reactions were performed to discriminate four *Babesia* spp.

### Specificity and sensitivity of the assay

**Fig. 2:**
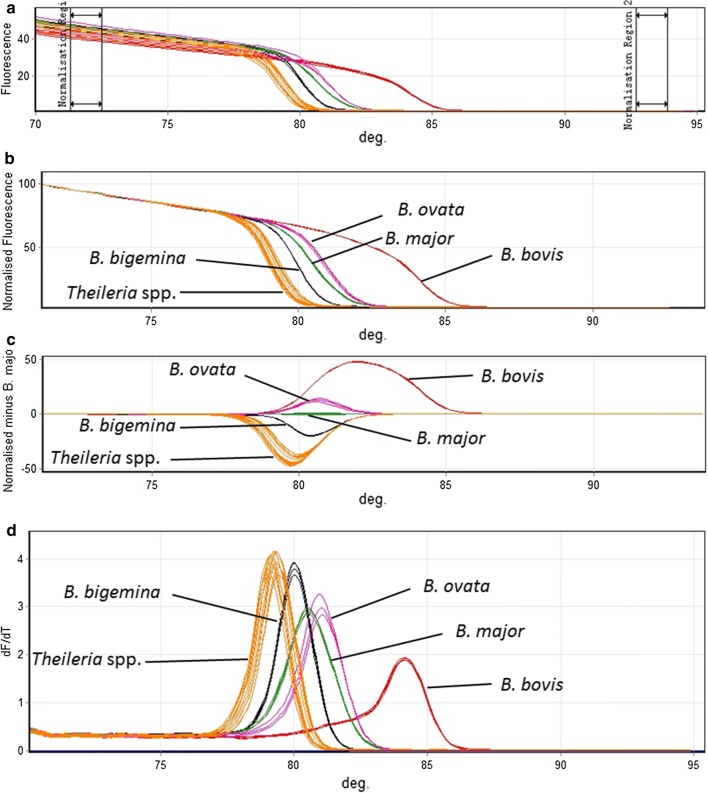
Detection and discrimination of the four *Babesia* spp. and *Theileria* spp. **a** Raw data from melt curve analysis. **b** Normalized HRM plots for *18S* rRNA amplicon; each sample was performed in duplicates in three independent experiments. **c** Normalized difference curves. **d** Derivative melting curve peaks

A primer pair (Bovis-B-7F and Bovis-B-7R) was designed to amplify DNA of all four *Babesia* spp. using the RT-PCR-HRM. However, there were cross-reactions with *T. annulata*, *T. orientalis* and *T. sinensis.* According to melting profiles and Tm values, the generated amplicon distinguished these species into five groups: *Theileria* spp., *B. bovis*, *B. bigemina*, *B. ovata* and *B. major*. The approach was not suitable for discriminating between species of the genus *Theileria* because no differences in melting profiles and Tm values were observed between *T. annulata*, *T. orientalis* and *T. sinensis* (Fig. 2). There was no amplification plot using genomic DNA isolated from bovine free-piroplasm infection and *A. marginale*.

**Fig. 3:**
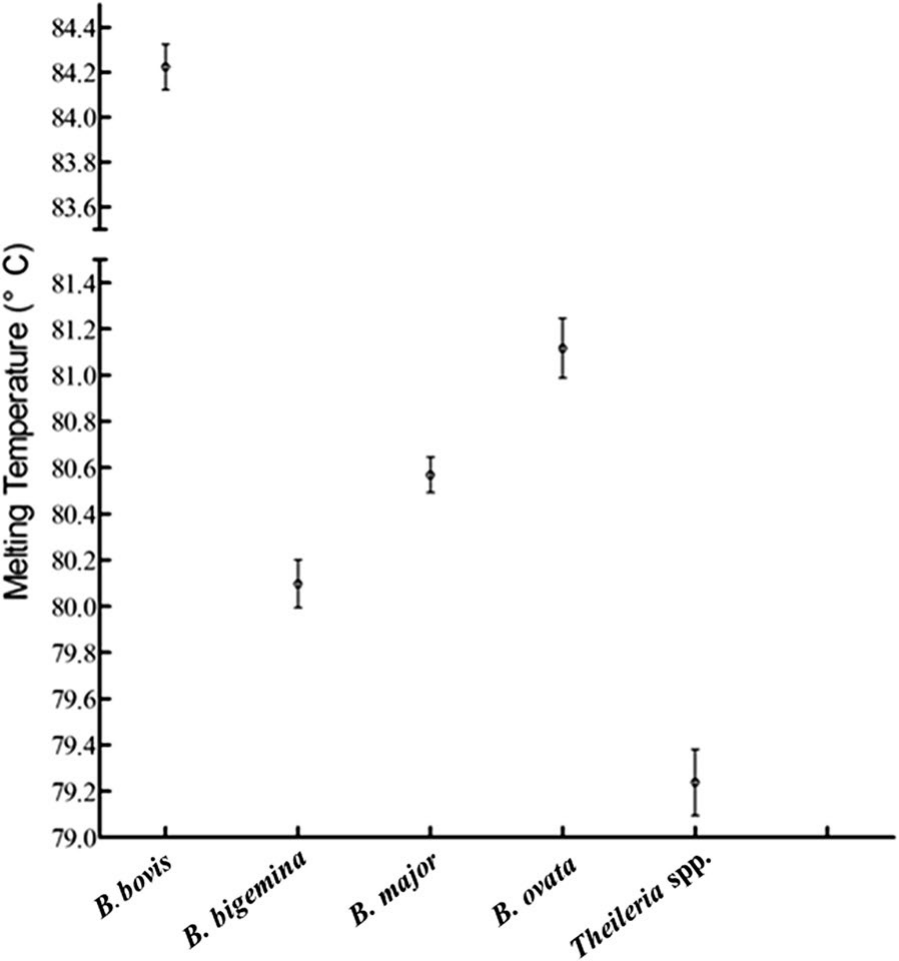
Tm values from the HRM analysis targeting the *18S* rRNA amplicon using a variable amount of initial DNA as a template. Each point represents the average and standard deviation of the Tm values, measured in duplicate

To evaluate the detection limit of the RT-PCR-HRM assay, 10 times serial dilutions from 10^7^ to 1 copy number/μl plasmid DNA containing target sequences of each *Babesia* spp. were performed in duplicate. The detection limit was one copy per reaction for each *Babesia* species. Standard curves for quantification assays, as confirmed by duplicates showed good correlations and efficiencies varying from 93.4 to 100.4% for all *Babesia* spp., with ranges of 10^1^ to 10^7^ copies (Additional file 1: Figure S1). The standard deviation and average of the melting temperature (Tm) of the amplicon were determined using variable DNA concentrations as template, isolated from each *Babesia* spp. infective to bovines. The average Tm values and their standard deviations for the *18S* rRNA amplicon of each *Babesia* spp. are presented in Table 1 and Fig. 3.

**Table 1:** Tm values of average melting curve peak for each *Babesia* spp.

Species	Tm value range (mean Tm ± SD) (°C)	
	DNA^a^	Variable amount of DNA^b^
*B. bovis*	84.15 ± 0.04	84.16 ± 0.19
*B. bigemina*	80.02 ± 0.02	80.04 ± 0.12
*B. major*	80.54 ± 0.03	80.62 ± 0.15
*B. ovata*	81.02 ± 0.05	81.24 ± 0.25
*Theileria* spp.	79.20 ± 0.19	79.24 ± 0.24

^a^The Tm values obtained from HRM using 20 ng of genomic DNA in 3 repeat reactions in 3 independent experiments

^b^The Tm values obtained from HRM using a variable amount of genomic DNA ranging from 5 to 50 ng in 3 repeat reactions in 3 independent experiments

### Evaluation of the RT-PCR-HRM assay using experimental and clinical samples

The RT-PCR-HRM assay for *18S* rRNA gene amplicon was also evaluated using genomic DNA extracted from cattle experimentally infected with *B. bovis* and *B. bigemina*, respectively. On the basis of melting curves and Tm values, species identified herein using the HRM analysis were 100% consistent with previous results (Table 2).

**Table 2:** Comparison of detection and discrimination results for *Babesia* spp. in experimentally infected animal samples and field samples

Sample source	Diagnostic method	
	HRM assay	nPCR assay and *18S* rRNA gene sequencing
Experimentally infected samples	*B. bovis* (*n* = 25)	*B. bovis* (*n* = 25)
	*B. bigemina* (*n* = 25)	*B. bigemina* (*n* = 25)
Field samples	*B. bovis* (*n* = 31)	*B. bovis* (*n* = 33)
	*B. bigemina* (*n* = 20)	*B. bigemina* (*n* = 20)
	*B. major* (*n* = 4)	*B. major* (*n* = 4)
	*B. ovata* (*n* = 5)	*B. ovata* (*n* = 5)
	*B. bovis *+* B. bigemina* (*n* = 3)	*B. bovis *+* B. bigemina* (*n* = 3)
	*B. bovis *+* B. ovata* (*n* = 2)	*B. bovis *+* B. ovata* (*n* = 2)
	*B. bigemina *+* B. ovata* (*n* = 1)	*B. bigemina *+* B. ovata* (*n* = 1)
	*B. bovis *+* B. major* (*n* = 1)	*B. bovis *+* B. major* (*n* = 1)
	*B. bovis *+* B. bigemina *+* B. ovata* (*n* = 1)	*B. bovis *+* B. bigemina *+* B. ovata* (*n* = 1)
	*B. bovis*, *B. bigemina *+* B. major* (*n* = 0)	*B. bovis *+* B. bigemina *+* B. major* (*n* = 1)

**Fig. 4:**
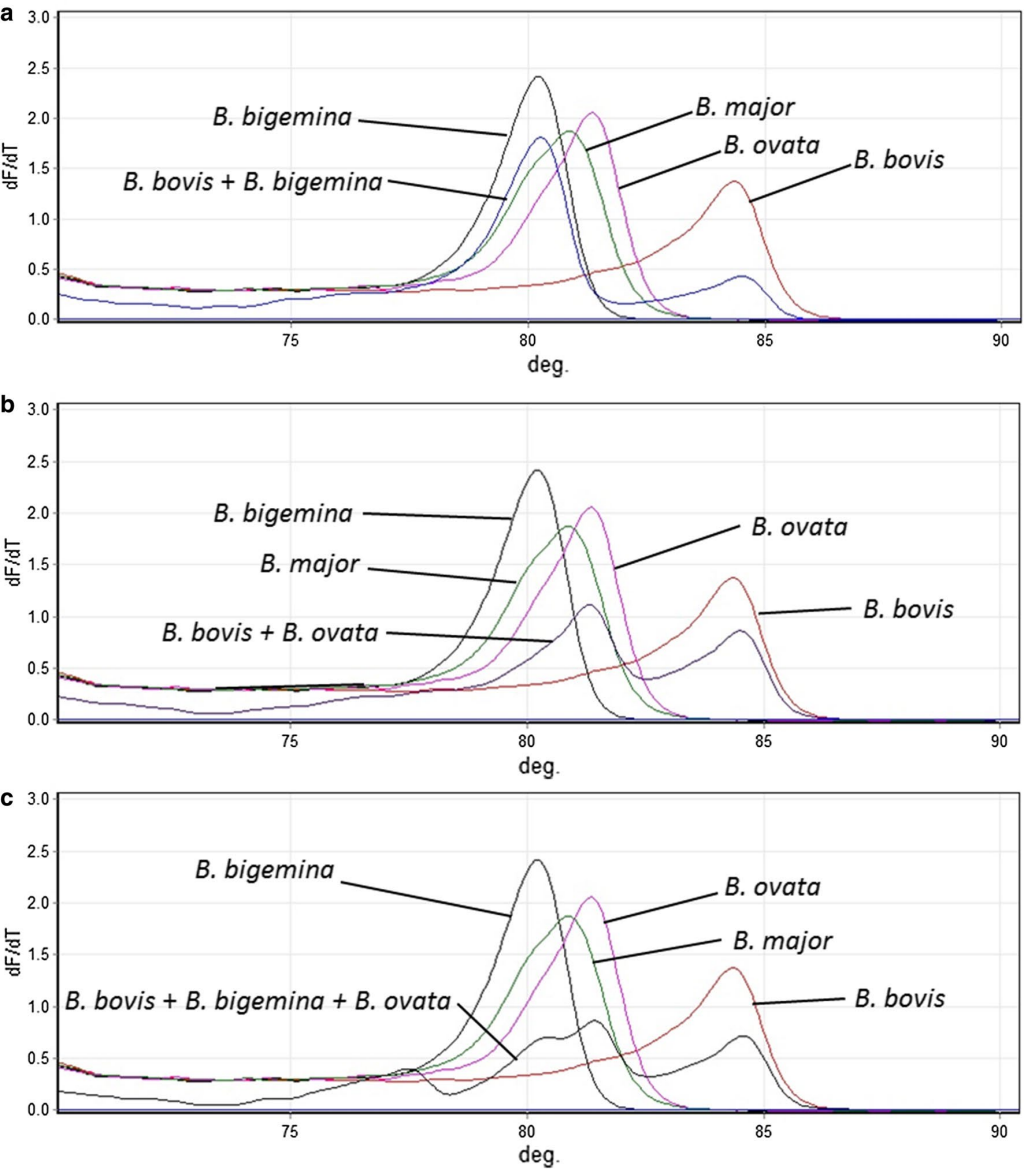
Detection and discrimination of mixed infectious clinical samples. **a** *Babesia bovis* + *B. bigemina* co-infection. **b** *Babesia bovis* + *B. ovata* co-infection. **c** *Babesia bovis* + *B. bigemina* + *B. ovata* triple infection

**Fig. 5:**
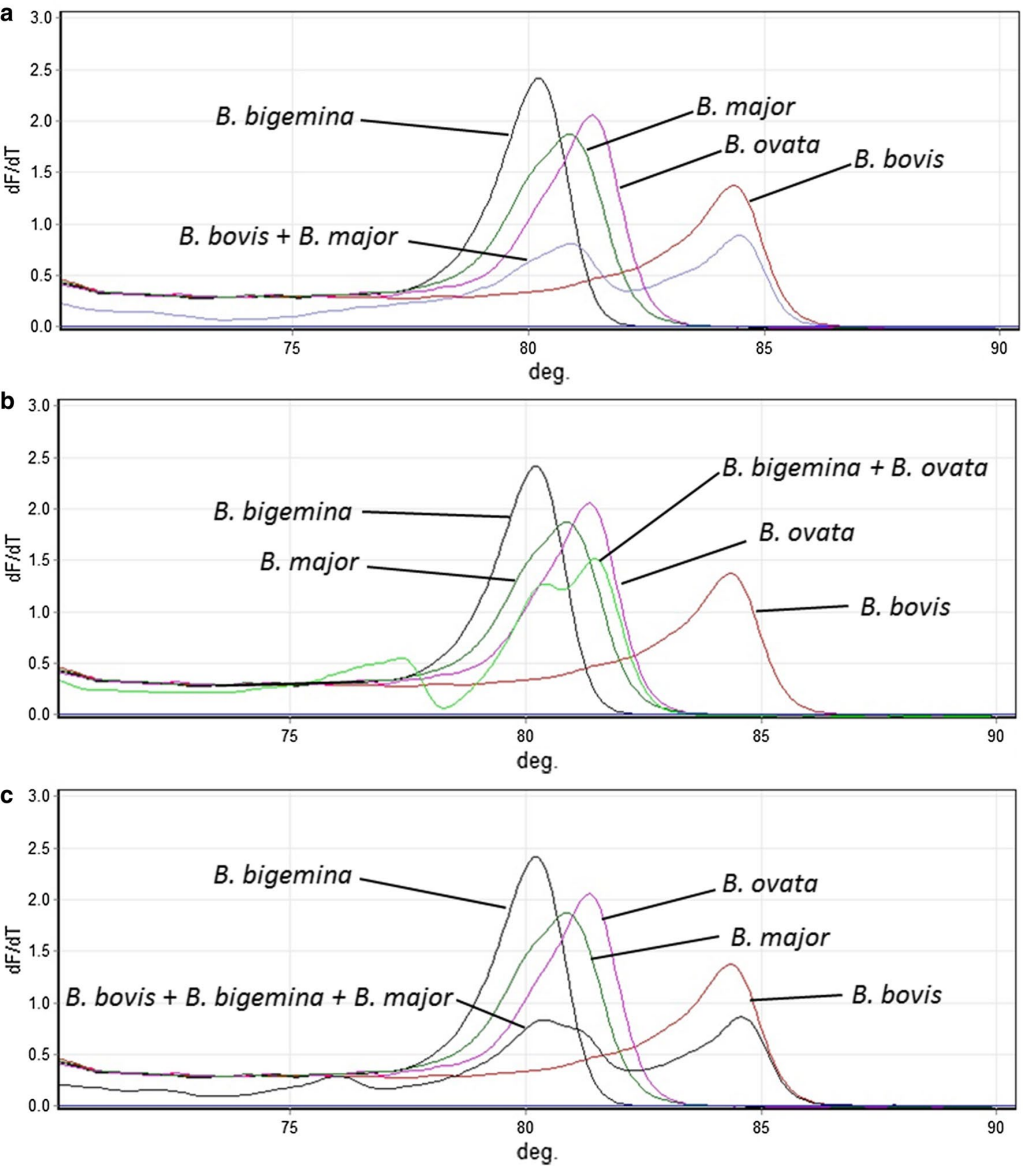
Detection and discrimination of mixed infectious clinical samples. **a** *Babesia bovis* + *B. major* co-infection. **b** *Babesia bigemina* + *B. ovata* co-infection. **c** *Babesia bovis* + *B. bigemina* + *B. major* triple infection

From the screening of clinical samples, 33 were identified as *B. bovis* infections. However, there were no amplifications for two clinical samples using the RT-PCR HRM assays. For the remaining samples, the HRM assays results were in agreement with the results, determined by nested PCR analysis and gene sequencing (Table 2). This approach was also suitable for the detection and discrimination of double and triple infections, except for mixed infections for *B. bovis*, *B. bigemina* and *B. major*, which only generated two peaks, observed on the melting curve plot (Figs. 4, 5). Moreover, the Tm values of the peaks were ambiguous.

## Discussion

Each *Babesia* spp. infective to bovines has different contribution to economic losses of livestock industry. For example, *B. bovis* and *B. bigemina* are the major causative agents of bovine babesiosis in China but *B. ovata* and *B. major* are considered as benign *Babesia* spp. which cause mild anemia or subclinical signs [3, 35]. Therefore, rapid detection and accurate discrimination of *Babesia* spp. are critical to clinical diagnosis and epidemiological study. Microscopy is considered to be the gold standard for babesiosis confirmation [16]. However, this approach has several limitations: it is time consuming, has a low sensitivity and is unable to distinguish species, which ultimately requires a combined approach with other diagnostic methods [17, 18]. Due to its high sensitivity, specificity, time efficiency and reproducibility, PCR based methods are predominately used to identify and distinguish *Babesia* species [8, 19–21]. However, these assays require the subsequent manipulation of the PCR product, which may introduce amplicon contamination [36]. HRM analysis is an alternative method for the simultaneous detection and discrimination of species. Based on slight differences in nucleotide composition, specific double-stranded DNA dissociation curve profiles and Tm values can be generated to discriminate *Babesia* spp. The HRM methodology has been employed to identify *Theileria equi* and *Babesia caballi* and to differentiate *Babesia* spp. infecting humans and dogs [28, 29]. Recently, Chua et al. [27] explored HRM analysis for the simultaneous detection of all five human *Plasmodium* spp. These available results indicated that HRM treating the *18S* rRNA gene is an attractive approach for the detection and differentiation of species.

Here, we successfully developed a HRM assay for the rapid detection and differentiation of four *Babesia* spp. circulating in Chinese bovine herds. Targeting the *Babesia* spp. *18S* rRNA gene, a primer pair was designed to amplify an amplicon containing polymorphic regions in different species and conserved intra-species. The HRM approach generated melting profiles and Tm values, which can be used to discriminate *B. bovis*, *B. bigemina*, *B. major* and *B. ovata*.

Initial DNA template concentrations were found to impact Tm fluctuations, which had previously been reported [37, 38]. Once Tm values and melting curves shape are shifted, it can lead to a misidentification of specific species. In our analysis, DNA concentration slightly influenced Tm values for all four *Babesia* spp., only exceeding 0.2 °C for *B. ovata*. For reliable differentiation, only Tm values exceeding 0.25 °C were considered as different species. The four species that did not present an overlapping Tm range and melting curve profiles were *B. bovis*, *B. bigemina*, *B. major* and *B. ovata*, which could be accurately discriminated by the HRM analysis. In addition, all available *18S* rRNA sequences from the NCBI database were aligned and screened for interspecific and intraspecific polymorphisms. The theoretical Tm values and sequence similarities of these four *Babesia* spp. isolated from different geographical regions were predicted by the *in silico* analyses using Oligo Calculator v.3.27 [39]. Of the isolates, two strains of *B. ovata* and one of *B. bovis* presented theoretical Tm values whose differences exceeded 0.25 °C, which could be the result of real sequence polymorphisms, sequencing errors or reflect different taxa (Additional file 2: Table S1).

Blood samples comprising both field-collected samples and samples from experimentally infected cattle were used to evaluate and compare HRM with the identification results using nested PCR and *18S* rRNA gene sequencing. Not all amplicons were successfully amplified for all the samples using the RT-PCR HRM approach; this could be due to differences in sensitivity of the diagnostic assay and the low parasitemia may have led to lack of amplification for these field-collected samples. The double or triple species co-infection was also accurately distinguished by HRM in this study. Multiple melting curve peaks, generated by combinational species, could be presented on the derivative melting curve profiles of samples infected with *Babeisa* spp. The melting curve shapes from field samples could be accurately distinguished *Babesia* spp. infective to cattle, when standard positive controls were included in each independent analysis.

## Conclusions

The RT-PCR-HRM analysis developed in this study is a promising tool for the detection and discrimination of *Babesia* spp., the causative agents of bovine babesiosis in China. There are no requirements for further manipulation of PCR products, such as gel fractionation, restriction fragment length polymorphism analysis and gene sequencing, thereby avoiding PCR cross-contamination in the laboratory.

## Supplementary information

**Additional file 1: Figure S1.** Efficiency and correlation of *18S* rRNA amplicon real-time PCR for DNA isolated from four *Babesia* spp. Standard curves were derived from a plasmid bearing the *18S* rRNA gene from *B. bovis* (**a**), *B. bigemina* (**b**), *B. major* (**c**) and *B. ovata* (**d**). A 10-fold dilution of the plasmid, ranging from 10^7^ to 10^1^ copies per reaction, was used as a template and performed in duplicate.

**Additional file 2: Table S1.** Sequence analysis of *18S* rRNA amplicons from four *Babesia* spp. The nucleotide sequences containing the *18S* rRNA amplicon were retrieved from the GenBank database.

## Data Availability

Not applicable.

## Abbreviations

RT-PCR-HRM: Real-time PCR high resolution melting analysis
VVBD: Vectors and Vector-Borne Diseases Laboratory
LVRI: Lanzhou Veterinary Research Institute
Tm: melting temperature
Cq: threshold cycle number
PCR: polymerase chain reaction
DNA: deoxyribonucleic acid
dsDNA: double stranded DNA
