# Supplementary figures and images for: High resolution melting analysis of the 18S rRNA gene for the rapid diagnosis of bovine babesiosis
Source: Parasit Vectors. 2019 Nov 6;12:523. doi: 10.1186/s13071-019-3781-4 (PMC6833191; doi:10.1186/s13071-019-3781-4)

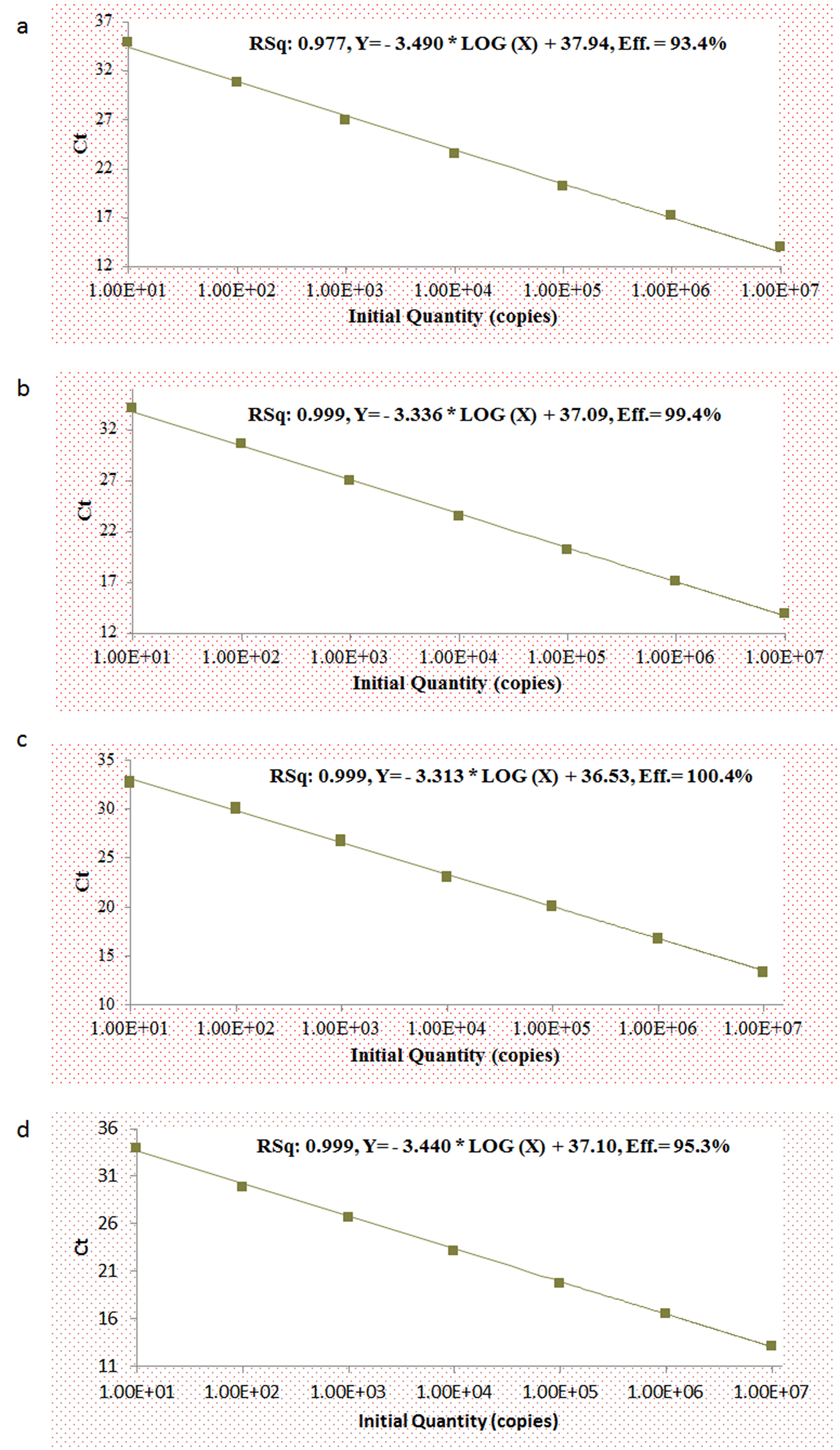

Supplement: Supplementary file 1 — Additional file 1: Figure S1. Efficiency and correlation of 18S rRNA amplicon real-time PCR for DNA isolated from four Babesia spp. Standard curves were derived from a plasmid bearing the 18S rRNA gene from B. bovis (a), B. bigemina (b), B. major (c) and B. ovata (d). A 10-fold dilution of the plasmid, ranging from 107 to 101 copies per reaction, was used as a template and performed in duplicate. [file 13071_2019_3781_MOESM1_ESM.tif]
