# Supplementary material for: High resolution melting analysis of the 18S rRNA gene for the rapid diagnosis of bovine babesiosis
Source: Parasit Vectors. 2019 Nov 6;12:523. doi: 10.1186/s13071-019-3781-4 (PMC6833191; doi:10.1186/s13071-019-3781-4)
Supplement: Supplementary file 2 — Additional file 2: Table S1. Sequence analysis of 18S rRNA amplicons from four Babesia spp. The nucleotide sequences containing the 18S rRNA amplicon were retrieved from the GenBank database. [file 13071_2019_3781_MOESM2_ESM.docx]

**Additional file 2: Table S1.** Sequence analysis of *18S* rRNA amplicons from four *Babesia* spp. The nucleotide sequences containing the 18S rRNA amplicon were retrieved from the GenBank database

| Species | Isolate site/strain | Accession | Amplicon of *18S* rRNA | |
| --- | --- | --- | --- | --- |
|  |  |  | **Similarity Index** | **Theoretical Tm (°C)** |
| B. ovata | China/Dongdatan 65 | KX870098 | 1.000 | 81.15 |
| *B. ovata* | China/Lushi | AY603401 | 1.000 | 81.15 |
| *B. ovata* | Korea | AY081192 | 1.000 | 81.15 |
| *B. ovata* | Japan/Miyake | LC125457 | 0.967 | 79.87 |
| *B. ovata* | China/Zhangjiachuan | AY603400 | 0.967 | 79.87 |
| *B. major* | France/France 1 | EU622907 | 1.000 | 80.87 |
| *B. major* | France/Mac30 | GU194290 | 1.000 | 80.87 |
| *B. major* | China/Yilli | AY603399 | 1.000 | 80.87 |
| *B. major* | Italy/PAR02_09_9coltCryFc | JF802039 | 1.000 | 80.87 |
| *B. major* | Italy/PAR 02_09-13coltF | JF802040 | 1.000 | 80.87 |
| *B. bovis* | South Africa/vaccine | L19078 | 1.000 | 82.87 |
| *B. bovis* | Mexico/B_bo18 | EF458218 | 1.000 | 82.87 |
| *B. bovis* | Brazil/BRC01 | FJ426364 | 1.000 | 82.87 |
| *B. bovis* | USA/USDA IA clone 4 | HQ264111 | 1.000 | 82.87 |
| *B. bovis* | USA/USDA IA clone 6 | HQ264112 | 1.000 | 82.87 |
| *B. bovis* | Australia: Queensland/ 8284 Dixie | JQ437260 | 1.000 | 82.87 |
| *B. bovis* | Australia: Queensland/ H81 | JQ437262 | 1.000 | 82.87 |
| *B. bovis* | China: Hunan | JQ723013 | 1.000 | 82.87 |
| *B. bovis* | China/boLushi | JX495403 | 1.000 | 82.87 |
| *B. bovis* | India/Bareilly | KF928959 | 1.000 | 82.87 |
| *B. bovis* | vaccine | L19077 | 1.000 | 82.87 |
| *B. bovis* | Australia/B_bo03 | EF458210 | 1.000 | 82.87 |
| *B. bovis* | Australia/B_bo05 | EF458211 | 1.000 | 82.87 |
| *B. bovis* | Brazil/B_bo07 | EF458212 | 1.000 | 82.87 |
| *B. bovis* | Brazil/B_bo08 | EF458213 | 1.000 | 82.87 |
| *B. bovis* | Brazil/B_bo09 | EF458214 | 1.000 | 82.87 |
| *B. bovis* | Brazil/B_bo10 | EF458215 | 1.000 | 82.87 |
| *B. bovis* | USA: Texas/B_bo16 | EF458216 | 1.000 | 82.87 |
| *B. bovis* | Mexico/B_bo17 | EF458217 | 1.000 | 82.87 |
| *B. bovis* | Portugal | AY150059 | 0.992 | 82.58 |
| *B. bigemina* | China:Guangdong | JQ723014 | 1.000 | 79.82 |
| *B. bigemina* | China/493 | HQ840959 | 1.000 | 79.82 |
| *B. bigemina* | China/563 | HQ840960 | 1.000 | 79.82 |
| *B. bigemina* | China/biLushi | JX495402 | 1.000 | 79.82 |
| *B. bigemina* | Brazil/BRC02 | FJ426361 | 1.000 | 79.82 |
| *B. bigemina* | Uganda/MT22 | KU206292 | 1.000 | 79.82 |
| *B. bigemina* | Uganda/MT23 | KU206293 | 1.000 | 79.82 |
| *B. bigemina* | Uganda/MT24 | KU206294 | 1.000 | 79.82 |
| *B. bigemina* | Uganda/KT4 | KU206295 | 1.000 | 79.82 |
| *B. bigemina* | Uganda/MT25 | KU206296 | 1.000 | 79.82 |
| *B. bigemina* | Uganda/MT26 | KU206297 | 1.000 | 79.82 |
| *B. bigemina* | Uganda/MT21 | KU206291 | 1.000 | 79.82 |
| *B. bigemina* | Turkey/Trkoz10 | KP745623 | 1.000 | 79.82 |
| *B. bigemina* | Turkey/Trkene3yumurta | KP745624 | 1.000 | 79.82 |
| *B. bigemina* | Australia/RG | JQ437264 | 1.000 | 79.82 |
| *B. bigemina* | Australia/B_bi02 | EF458191 | 1.000 | 79.82 |
| *B. bigemina* | Australia/B_bi03 | EF458192 | 1.000 | 79.82 |
| *B. bigemina* | Australia/B_bi04 | EF458193 | 1.000 | 79.82 |
| *B. bigemina* | Australia/B_bi05 | EF458194 | 1.000 | 79.82 |
| *B. bigemina* | Zimbabwe/B_bi06 | EF458195 | 1.000 | 79.82 |
| *B. bigemina* | Brazil/B_bi07 | EF458196 | 1.000 | 79.82 |
| *B. bigemina* | Brazil/B_bi08 | EF458197 | 1.000 | 79.82 |
| *B. bigemina* | Kenya/ B_bi11 | EF458200 | 1.000 | 79.82 |
| *B. bigemina* | Mexico/B_bi12 | EF458201 | 1.000 | 79.82 |
| *B. bigemina* | Uruguay/B_bi14 | EF458202 | 1.000 | 79.82 |
| *B. bigemina* | Mexico/B_bi16 | EF458203 | 1.000 | 79.82 |
| *B. bigemina* | Mexico/B_bi17 | EF458204 | 1.000 | 79.82 |
| *B. bigemina* | Puerto Rico/B_bi18 | EF458205 | 1.000 | 79.82 |
| *B. bigemina* | Virgin Islands/B_bi19 | EF458206 | 1.000 | 79.82 |
| *B. bigemina* | India/Umiam | KF112076 | 1.000 | 79.82 |
| *B. bigemina* | Spain/Spain_1 | DQ785311 | 1.000 | 79.82 |
| *B. bigemina* | Switzerland/Swiss_6 | KM046917 | 1.000 | 79.82 |
| *B. bigemina* | Turkey/B_bi10 | EF458199 | 1.000 | 79.82 |
| *B. bigemina* | Brazil/B_bi09 | EF458198 | 0.992 | 79.84 |
| *B. bigemina* | USA/H7 clone 2 | HQ264113 | 1.000 | 79.82 |
| *B. bigemina* | USA/H7 clone 9 | HQ264114 | 1.000 | 79.82 |
| *B. bigemina* | USA/H7 clone 6 | HQ264115 | 1.000 | 79.82 |
| *B. bigemina* | USA/H7 clone 7 | HQ264116 | 1.000 | 79.82 |
| *B. bigemina* | USA/H7 clone 8 | HQ264117 | 1.000 | 79.82 |
| *B. bigemina* | USA/H7 clone 92 | HQ264118 | 1.000 | 79.82 |
